# Analysis of outer hair cell electromechanics reveals power delivery at the upper-frequency limits of hearing

**DOI:** 10.1098/rsif.2022.0139

**Published:** 2022-06-08

**Authors:** Richard D. Rabbitt

**Affiliations:** Biomedical Engineering, Otolaryngology, and Neuroscience Program, University of Utah, 36 S. Wasatch Drive, SMBB 3100, Salt Lake City, UT 84112, USA

**Keywords:** electromotility, prestin, cochlea, piezoelectricity, biological motor, imaginary capacitance

## Abstract

Outer hair cells are the cellular motors in the mammalian inner ear responsible for sensitive high-frequency hearing. Motor function over the frequency range of human hearing requires expression of the protein prestin in the OHC lateral membrane, which imparts piezoelectric properties to the cell membrane. In the present report, electrical power consumption and mechanical power output of the OHC membrane–motor complex are determined using previously published voltage-clamp data from isolated OHCs and membrane patches. Results reveal that power output peaks at a best frequency much higher than implied by the low-pass character of nonlinear capacitance, and much higher than the whole-cell resistive–capacitive corner frequency. High frequency power output is enabled by a −90° shift in the phase of electrical charge displacement in the membrane, manifested electrically as emergence of imaginary-valued nonlinear capacitance.

## 1. Introduction

The sensitivity and frequency bandwidth of mammalian hearing relies on active amplification of sound-induced vibrations in the cochlea by electro-motile outer hair cells (OHCs), which change length in response to changes in membrane potential [1]. Over the frequency range of human hearing, voltage-driven electro-motility is imparted by expression of the transmembrane protein prestin [2,3] through a mechanism that couples electrical charge displacement in the prestin–membrane motor complex to changes in cell length. The structure of prestin [4–6] suggests OHC piezoelectricity arises on the whole-cell level from a large number of prestin molecules undergoing conformational changes on the nanoscale, with each conformational change involving an electrical charge displacement and a change in membrane area. But this mechanism might not extend beyond the range of human hearing to species with ultrasonic hearing. Behavioural evidence in mice suggests prestin expression is not required for hearing above 40 kHz [7]. Although the high frequency mechanism(s) remains unknown, it is possible the lipid bilayer itself or membrane–protein interactions are involved [8]. Lipid bilayer flexoelectricity [9] putatively underlies voltage-dependent force generation in cell membrane tethers [10] and mechanical deformation of axons during electrical activity [11,12], but it is not known if flexoelectricity contributes to ultrasonic OHC function. It has been shown that electro-mechanical coupling on the whole-cell level obeys Maxwell reciprocity [13,14], as expected if the Gibbs free energy governing OHC electromechanics is piezoelectric [15,16].

There are conflicting reports addressing the speed of the OHC motor. Optical coherence tomography measurements in the living cochlea support the cycle-by-cycle amplification hypothesis at frequencies up to at least 20 kHz in mouse [17], with amplification occurring primarily near the travelling wave peak in gerbil and mouse [18,19]. Analysis of power output by OHCs based on vibrations of the cochlear partition [20] provide additional evidence supporting the cycle-by-cycle amplification hypothesis. Evidence at odds with this view include the low-pass character of OHC displacement in echolocating bats [21] and second-order distortion products in gerbil [22]. Low-pass characteristics are also present in nonlinear capacitance (NLC) recorded in isolated membrane patches [23–25], and in voltage-driven displacement of isolated OHCs measured using the micro-chamber approach [26,27]. Precisely how the OHCs amplify vibrations in the cochlea at high frequencies given these low-pass features of the motor remains unknown.

Computational models of cochlear mechanics that treat OHCs as cycle-by-cycle piezoelectric actuators [28] replicate experimental data reasonably well over a broad bandwidth, including changes in vibrational patterns from apex to base [29]. Theoretical analysis of isolated OHCs driving a mechanical load further support the hypothesis that OHCs inject power into cochlear viscous load cycle-by-cycle over the entire auditory frequency bandwidth [30,31]. These cycle-by-cycle computational and theoretical considerations are consistent with cochlear amplification in whales and bats at frequencies greater than 100 kHz, but upon first inspection seem at odds with the low-pass characteristics of the motor noted above.

The present report re-examines data from isolated membrane patches and whole cells under voltage clamp conditions to determine if the data support the hypothesis that power output and electro-mechanical power conversion are low pass. Results reject the null hypothesis and demonstrate that electro-mechanical power conversion is ultrafast, and generates mechanical power output up to the highest frequencies tested to date. Peak power output occurs at frequencies much higher than might be expected on the basis of traditional measures of NLC or voltage-driven cell displacement. Results further support the hypothesis that power output in isolated membrane patches and OHCs is band-pass and dependent on the mechanical load, similar to that measured in the cochlea [19] and predicted previously on theoretical grounds for isolated cells [30,31].

## 2. Methods

### 2.1. Electrical power consumed under ideal voltage clamp

To find the electrical power consumed by the prestin–membrane motor complex for small sinusoidal signals under voltage-clamp conditions, the real-valued time-domain transmembrane voltage *v*(*t*), current *i*(*t*) and electrical charge displacement *q*(*t*) are written in terms of the frequency-domain counterparts using $v(t)=1/2(Ve^{\;j\omega t}+V^{*}e^{-j\omega t})$, $i(t)=1/2(Ie^{\;j\omega t}+I^{*}e^{-j\omega t})$ and $q(t)=1/2(Qe^{\;j\omega t}+Q^{*}e^{-j\omega t})$, respectively [32]. Upper case denotes the frequency domain, the * denotes the complex conjugate and *ω* is frequency in radians per second. The charge *Q* is equal to the capacitance *C* times voltage *V*, where *C* is the sum of the linear capacitance *C^L^* and the prestin-dependent nonlinear capacitance *C^N^*. Multiplying the time-domain electrode current *i*(*t*) times voltage *v*(*t*) and taking the time average gives the electrical power delivered by the patch pipette as $P_{i}=(1/2)IV^{*}$. Multiplying the membrane electrical displacement current d*q*/d*t* by voltage *v*(*t*) and taking the time average gives the electrical power *P_E_* consumed by the prestin motor complex:

2.1 $$P_{E}=\frac{-\omega }{2}Im(C^{N})V^{2},$$

which, by the Second Law of Thermodynamics, must be converted to heat plus mechanical power output by the motor. Notably, *P_E_* is completely independent of the real part of the nonlinear capacitance, demonstrating that *Re*(*C^N^*) is not related to mechanical power output by the motor complex in membrane patches or in OHCs [31].

### 2.2. Electrical power consumed under general conditions

To determine the electrical power consumed from experimental data under more general conditions, the OHC is treated as a single electrical compartment, where the voltage and current are related by Kirchhoff's current law: $(dq/dt)+i_{i}=i$. The input current from mechano-electrical transduction (MET) channels or recording electrode is *i* and the total ionic channel conduction current is *i_i_*. Under isothermal conditions, the charge displacement depends on voltage *v* and mechanical strain *s*. From the chain rule of calculus $dq/dt=c^{l}(dv/dt)+c^{s}(ds/dt)$, where $c^{l}=\partial q/\partial v$ is the capacitance voltage susceptibility (conventional ‘linear’ electrical capacitance) and $c^{s}=\partial q/\partial s$ is the capacitance strain susceptibility arising from the membrane motor complex [31,33]. It is important to note that *c^s^* arises from the chain rule and is agnostic to the specific molecular origin(s) of capacitance strain susceptibility. The voltage *v* is the transmembrane voltage, which can arise from both intracellular and extracellular voltage modulation in the cochlear organ of Corti. The whole cell displacement *x* is related to the strain by *x* = ℓ*s*, where ℓ is cell length. For small perturbations from the resting state (*v_o_*, *i_o_*, *x_o_*), the Fourier transform of Kirchoff's current balance for small perturbations in the frequency domain gives:

2.2 $$C^{L}j\omega V+C^{S}j\omega X+I_{i}=I,$$

where $j=\sqrt{-1}$. The parameter *C^S^* is proportional to the capacitance strain susceptibility and quantifies the relationship between the electrical displacement current in the motor complex and the whole-cell axial displacement. *I_i_* describes the linearized ionic currents in the frequency domain, while *I* is the input current.

Electrical power is determined by multiplying equation (2.2) by the complex conjugate of voltage (V*) and taking half of the real part, which is equivalent to the time-domain method used to derive equation (2.1). The right-hand side gives the electrical power input *P_E_* via the MET channels or patch electrode:

2.3 $$P_{T}=\frac{1}{2}Re(IV^{*}),$$

which is the *input electrical power* available from cycle-by-cycle modulation of the voltage and the MET receptor current (or electrode current). V* is the transmembrane voltage modulation determined from potentials on both sides of the membrane, a distinction that is important in the organ of Corti versus recordings from isolated cells in the dish. The left-hand side gives the electrical power lost to ion-channels:

2.4 $$P_{I}=\frac{1}{2}Re(I_{i}V^{*}),$$

and the electrical power consumed by the motor complex:

2.5 $$P_{E}=\frac{-\omega C^{S}}{2}Im(XV^{*}).$$

Notably, from equation (2.5), the electrical power consumed by the motor complex is zero if the voltage modulation is in phase with the displacement, and maximum if the voltage is in phase with velocity. This fact means the power consumed depends on the mechanical load acting on the prestin–membrane motor complex, because the load alters the phase of the displacement *X* even if the voltage *V* is constant. The parameter *C^S^* varies with reference state (*v_o_*, *i_o_*, *x_o_*) and configuration of prestin, but like passive electrical capacitance *C^L^*, is constant for small sinusoidal perturbations in voltage, current and displacement about the reference state. *C^S^* can be determined experimentally in the frequency domain after blocking ion channels. The direct approach is to measure *V*, *I* and *X* and apply equation (2.2) to determine *C^S^*.

Although simple, the form of equation (2.5) is difficult to use experimentally because it requires measuring *V* and *X* at the same time. Measuring *P_E_* is simplified under voltage clamp (equation (2.1)), but from thermodynamics the two must be identical (*P_E_* from equation (2.1) must be equal to *P_E_* from equation (2.5)). Equivalency is demonstrated by writing the OHC displacement as a function of voltage and force: *X* = *X*(*V*, *F*) and expanding in a Taylor series to find the total capacitive current arising from the motor complex is *jωC^S^X* = *jω*(*C^V^V* + *C^F^F*), where the capacitance voltage susceptibility is $C^{V}=C^{S}(\partial X/\partial V)$ and the capacitance force susceptibility is $C^{F}=C^{S}(\partial X/\partial F)$. After blocking ion channels, *C^V^* can be measured in voltage clamp under constant force, and *C^F^* can be measured in force clamp under constant voltage. While *C^S^* is a constant independent of load, both *C^V^* and *C^F^* depend on the mechanical strain in the motor complex and therefore depend on how the cell is loaded. Under ideal voltage clamp, a command voltage *V* causes the cell to displace against the load imposed by the cell itself and the environment. When working against a load, the force is a frequency-dependent function of voltage (e.g. *F* = *TV*, where *T* is a transfer function). Combining terms, the total capacitive current is *jωC^N^V*, where *C^N^* = *C^V^* + *TC^F^*, and the power consumed by the motor is found to be exactly the same as equation (2.1). Application of equation (2.1) simply requires experimental measurement of *C^N^* and *V*, and is a valid approximation regardless of the molecular origin(s) of NLC. Equation (2.1) clearly has experimental advantages, but equation (2.5) offers more insight into power output and consumption by the motor complex because the capacitance displacement susceptibility *C^S^* is independent of load, while *C^N^* is frequency-dependent and changes with the load on the membrane under the conditions of the experiment.

### 2.3. Mechanical power output

The mechanical power output by the motor complex is time-average of force times velocity and can be written in the frequency domain as:

2.6 $$P_{PM}=\frac{-\omega }{2}Im(XF^{*}),$$

where *X* is the axial cell displacement and *F* is the axial somatic force. Equation (2.6) can be used to estimate power output irrespective of the biophysical origins of force, but it is informative to demonstrate how power output is related to electrical power consumed based on thermodynamics of electromechanics which, to first order, is piezoelectric. For a simple non-dispersive one-dimensional piezoelectric model of an OHC, the force is related to the displacement and the voltage by F=(1/κℓ)X+(δ/κ)V, where *κ* is the compliance per length, ℓ is the cell length and *δ* is the voltage- and strain-dependent piezoelectric strain coefficient. Substituting into equation (2.6) gives the piezoelectric prediction for mechanical power output:

2.7 $$P_{PM}\approx \frac{-\delta \omega }{2\kappa }Im(XV^{*}),$$

which is identical to equation (2.5) providing *C^S^* = *δ*/*κ* (which is the piezoelectric force coefficient) but derived from the mechanical versus electrical perspective. Hence, in the simple piezoelectric model electrical power consumed by the motor is equal to the mechanical power driving displacement of the cell and the mechanical load.

### 2.4. Mechanical power dissipated by the load

Power consumed by the prestin–membrane motor complex is partially dissipated by heat in the cell itself and partially transmitted to the extrinsic mechanical load, a load that varies systematically in the cochlea along the tonotopic map. Insight can be gained into OHC power output by my treating the cochlear load as a simple spring–mass–damper. From Newton's Second Law: *D^L^X* = *F^T^*, where *X* is displacement of the load and *F^T^* is the total force acting on the load (OHC generated force plus applied force). In the frequency domain, the operator *D^L^* is

2.8 $$D^{L}=K^{L}\left(1-{\left(\frac{\omega }{\omega_{0}}\right)}^{2}+\zeta^{L}{\left(\frac{\;j\omega }{\omega_{0}}\right)}^{n}\right),$$

where stiffness of the load and OHC combined is *K^L^*, the passive undamped natural frequency is *ω*_0_ and the nondimensional viscous drag coefficient is *ζ^L^*. Equation (2.8) uses a fractional derivative *n* to model the viscous load [34], which gives rise to a power-law frequency dependence of viscous dissipation [31]. For an isolated cell in the dish, *D^L^* describes intrinsic properties of the cell itself plus the media, and in the cochlea *D^L^* describes the addition of the intrinsic properties of the cell plus the cochlear load, with stiffness, frequency and damping parameters adjusted according to tonotopic location. Multiplying equation (2.8) by velocity and averaging over time gives the power consumed by the viscous load in the frequency domain:

2.9 $$P_{M}=\frac{c\omega^{1+n}}{2}\sin\left(\frac{n\pi }{2}\right)X^{2},$$

where the damping parameter $c=K^{L\;}\zeta^{L}/\omega_{0}^{n}$. For *n* = 1, which is the standard first approximation, equation (2.9) reduces to the well-known expression for power consumption by a viscous damper: $P_{M}=(c\omega^{2}/2)X^{2}$. As expected, mass and stiffness do not appear in equation (2.9) because they do not dissipate power. Total mechanical power output can easily be estimated from equation (2.9) based on whole-cell displacement in the frequency domain after estimating the viscous dissipation parameters *c* and *n*.

## 3. Results

### 3.1. Mechanical power output delivered to the dissipative load

**Figure 1.**
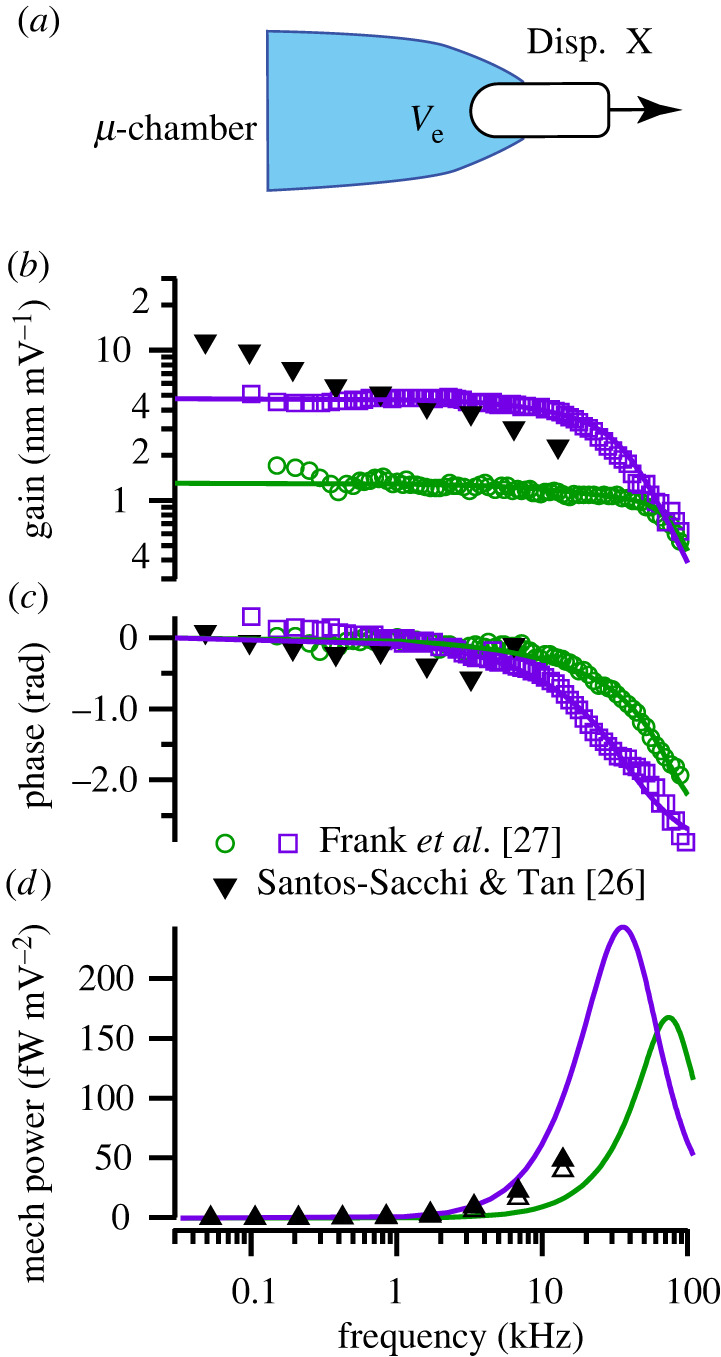
OHC mechanical power output in the dish. (*a*). Schematic of a micro-chamber used to apply sinusoidal voltage stimuli to the base of the cell with the OHC partially extending into media. (*b,c*) Magnitude and phase of the displacement *X* measured by Frank *et al*. (open symbols: open green circles, open purple squares) [27] and Santos-Sacchi & Tan (inverted triangles) [26]. Solid curves are curve-fits to equation (2.8) used to estimate the dissipation parameters ‘*c*’ and ‘*n*’. (*d*) Mechanical power output was determined from equation (2.9) and measured displacement *X*. Power output computed from the Frank *et al*. data peaked at frequencies as high as 74 kHz, when the phase of the displacement was −90° relative to the peak sinusoidal voltage. Power output computed from the Santos-Sacchi & Tan data (triangles: filled triangle, open triangle) was continuing to increase up to the highest frequency tested.

To determine frequency dependence of motor function in isolated OHCs, mechanical power output was determined from high-frequency voltage-driven displacement data reported by Frank *et al*. [27] and data reported by Santos-Sacchi & Tan [26]. Figure 1*a* illustrates the micro-chamber recording set-up, where electromotility was evoked by applying extracellular sinusoidal voltage commands *V_e_* to the base of OHCs. Results for two cells from Frank *et al*. are shown in figure 1 in the form of magnitude (*b*) and phase (*c*). Solid curves are fits to the overdamped spring–mass–damper given by equation (2.8) for the longer cell (purple, squares) and the shorter cell (green, circles), with fit parameters: *ω_o_* = (3.45 × 10^5^, 1.73 × 10^5^) rad s^−1^, *ζ^L^* = (1.43, 1.74) and *n* = (0.84, 0.90) respectively. Mechanical power output was found using equation (2.9), shown as the two solid curves in figure 1*d*, peaking at 34 kHz and 73 kHz for the two cells. OHC power output was maximum at the frequency when the displacement of the cell lagged the stimulus voltage by approximately 90°, which is the frequency where the force generated by the motor aligns with cell velocity. Using conventional damping *n* = 1 reduces goodness of the curve fits, but does not change the fact that peak power output occurs at frequencies well above the corner frequency of whole cell displacement. This occurs because the viscous drag force depends on velocity rather than displacement, causing the mechanical power output to reflect velocity squared, in contrast to displacement squared.

For low frequency voltage clamp recordings from OHCs in the dish, voltage is in phase with displacement, and power conversion by the prestin–motor complex is zero (equation (2.5)). Hence, low frequency electromotility is only indirectly related to OHC function as a motor. Significant power conversion only occurs at high frequencies as viscosity compels the cell displacement to shift approximately 90° relative to the voltage, maximizing electrical power consumption *P_E_* and mechanical power output *P_M_*.

In the Frank *et al*. experiments, the intracellular voltage was not measured, so the precise resting potential *v_o_* and cycle-by-cycle voltage modulation *v* were not known. This transmembrane voltage ambiguity likely explains why the isometric force recorded in the micro-chamber configuration (approx. 0.03 nN mV^−1^ [27]) was smaller than the isometric force recorded in whole-cell voltage clamp (approx. 0.10 nN mV^−1^ [35]). In subsequent micro-chamber experiments by Santos-Sacchi & Tan [26], an offset voltage was applied to restore the resting potential and increase electromotility gain to obtain summary data reproduced here in figure 1*b,c* (inverted triangles). Using the Santos-Sacchi & Tan data, equation (2.9) gives the mechanical power output in figure 1*d*. Power was computed directly from their data using the non-dimensional OHC viscous drag coefficient estimated for conventional viscous dissipation (*ζ^L^* = 1.43, *n* = 1, open triangles) and for power law viscous dissipation (*ζ^L^* = 1.43, *n* = 0.84, filled triangles), with both estimates giving almost identical results. Changing *ζ^L^* relative to the value found from the broader-bandwidth Frank *et al*. data change the magnitude of the power output curve but not the frequency dependence. Power output was continuing to increase in the Santos-Sacchi & Tan experiments up to the highest frequency tested (figure 1*d*, triangles), proving high frequency motor function and forcing rejection of the low-pass hypothesis.

Results in figure 1 are consistent with power delivered to a viscous load by a force-driven spring–mass–damper system. Based on simple mechanics, the frequency limit of OHC power output is not limited by the low-pass character of electrically driven whole-cell displacement but instead depends strongly on shortening velocity and is limited by the speed at which the force can displace the mechanical load. In the micro-chamber, the mechanical load on the OHC arises from the media and the cell itself. The frequency of maximum real power output is aligned with the best frequency where contributions of mass and stiffness nearly cancel. Ultimately, as computed from Frank *et al*. data (figure 1*d*), power output declines at high frequencies as expected from mechanics. The band-pass nature of OHC power output occurs in the micro-chamber because the cell stiffness limits power output at low frequencies, while the fluid and cell mass limit power output at high frequencies. In the cochlea, maximum power output would be expected to occur near the characteristic frequency where the mass and stiffness nearly cancel and the load is dominated by viscous drag [30,31].

### 3.2. Electrical power converted by the membrane–prestin motor complex ***P_P_***

**Figure 2.**
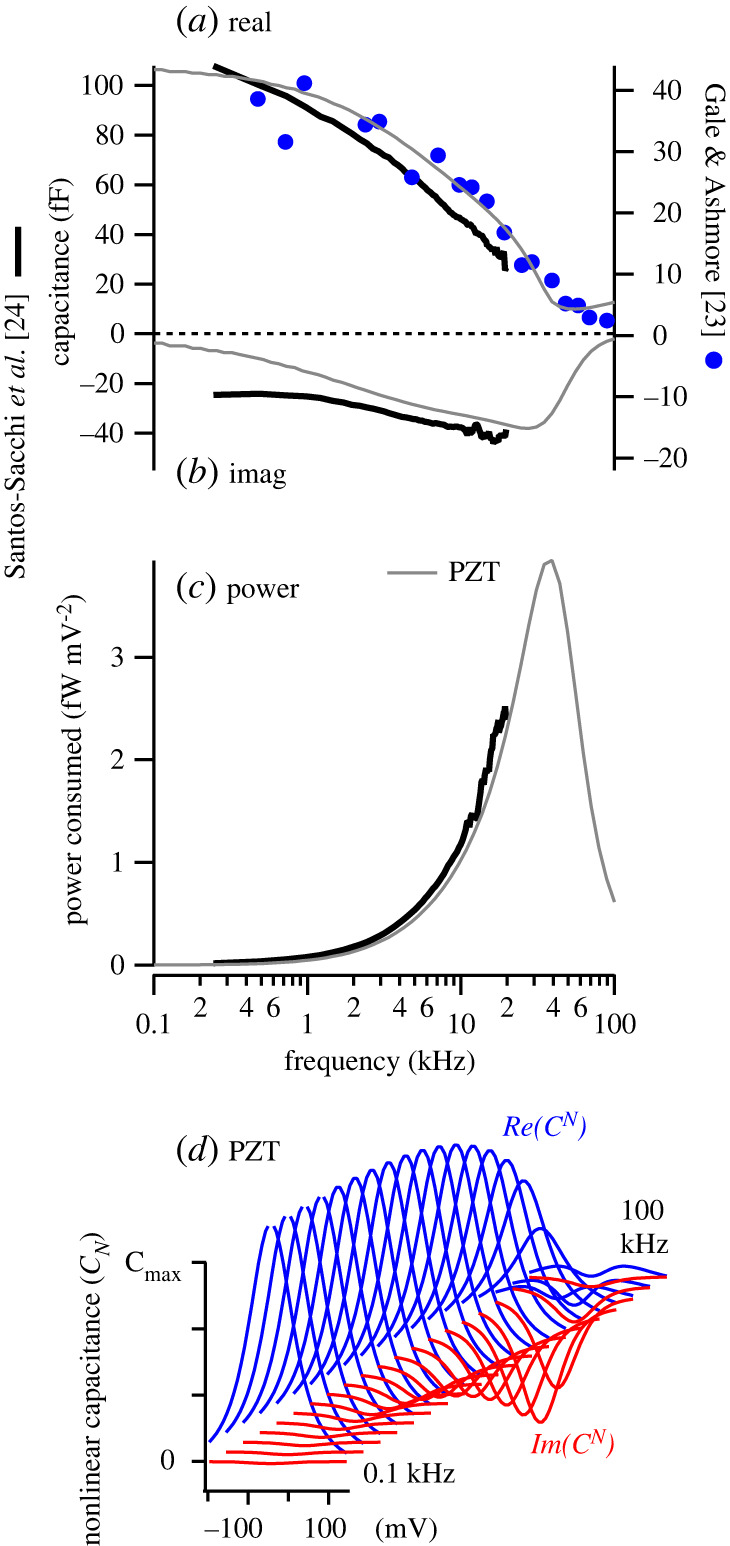
Membrane patch electrical power consumption. (*a*) Real NLC, *Re*(*C^V^*), recorded from OHC membrane macro-patches from data reported by Santos-Sacchi *et al*. (thick black) [24]. The right axis shows NLC recorded using an alternative approach by Gale & Ashmore (blue symbols) [23] for comparison. (*b*) Imaginary NLC, *Im*(*C^V^*), recorded from OHC membrane macro-patches from data reported by Santos-Sacchi *et al*. (thick black). (*c*) Electrical power consumed by the prestin-motor complex (thick black) computed using equation (2.6) and *Im*(*C^V^*) reported by Santos-Sacchi *et al*. (thick black, *b*). (*a–c*) Thin grey curves are predictions of PZT theory [31]. (*d*) Voltage and frequency dependence of PZT theory, with frequency-dependence of the peaks corresponding to the solid grey curves in panels *a–c*.

To determine the speed of the motor function in membrane patches, frequency-dependent electrical power consumption was determined directly from macro-patch NLC data reported and by Santos-Sacchi *et al*. [24], replotted in figure 2 as the real *Re*(*C^N^*) (*a*) and imaginary *Im*(*C^N^*) (*b*) components. For comparison, capacitance reported by Gale & Ashmore [23] is also shown (right axis, blue circles, real and imaginary components were not separated in the Gale & Ashmore report). The magnitude of the NLC between the two reports differs primarily due to the size of the patch. Both datasets clearly show the magnitude of *C^N^* declining with frequency (figure 2*a*). *Re*(*C^N^*) (figure 2*a*, thick black) has a low-pass characteristic beginning to roll off below 2 kHz and showing a power-law frequency dependence. But, as proven above (equations (2.1), (2.5)), the real component of NLC is not related to electro-mechanical power conversion and therefore has little to do with function as a motor. Instead, the electrical power consumed by the motor complex is proportional to negative frequency times the imaginary component of NLC. The Santos-Sacchi *et al*. data clearly show *Im*(*C^N^*) is indeed negative in prestin-expressing membrane patches (figure 2*b*, thick black), and electrical power consumed by the motor continues to increase in magnitude up to the highest frequency tested (figure 2*c*, thick black).

To interpret electrical power consumed in prestin expressing macropatches, we compared results in figure 2 to predictions of OHC piezoelectric theory (PZT [31]). Solid grey curves in figure 2*a, b* and *c* show theoretical predictions for *Re*(*C^N^*), *Im*(*C^N^*) and *P_P_* , respectively (computed at the voltage of peak *Re*(*C^N^*)). For parameters approximated from the Santos-Sacchi *et al*. data [24], PZT theory predicts band-pass power consumption to peak near 37 kHz (figure 2*c*, thin curve). Results support the hypothesis that electrical power consumed by the prestin–motor complex is band-pass and operates at frequencies much higher than would be implied by the low-pass character of *Abs*(*C^N^*), or *Re*(*C^N^*). Frequency and voltage dependence of *C^N^* predicted by PZT is illustrated in figure 2*d*. By the Second Law of Thermodynamics, the electrical power consumed by the membrane patch (figure 2*c*) must be delivered to dissipation inside the membrane itself and/or to viscosity of the fluid media surrounding the patch (figure 1*d*).

## 4. Discussion

Results in figure 2 demonstrate high-frequency electro-mechanical power conversion by prestin expressing membrane patches, but precisely where the power goes is a subject of debate with hypotheses differing dramatically between reports. In piezoelectric theory applied to isolated OHCs or membrane patches [31] almost all of the power is delivered to the viscous fluid media, and *Im*(*C_n_*) emerges with increasing frequency as viscosity compels the force from the motor to align with the mechanical rate of deformation. Electro-mechanical power conversion in PZT is band-pass, and is predicted to peak under macro-patch voltage clamp conditions near 30 kHz (figure 2*c*, thin grey). The peak occurs at the characteristic frequency of the patch where the load is dominated by viscosity (versus mass or stiffness). For the same reason, peak power output by OHCs in the cochlea is predicted by PZT to align with characteristic frequency [28,30,31,36]. By contrast, if the mechanical load is neglected, *Im*(*C_n_*) arises in transition-state theory from the rate constants [24], which means the conformation transition itself dissipates all of the power as heat, analogous to dielectric loss with no possibility of power delivery to cochlear amplifier.

Results in figure 1 offer compelling evidence that the electrical power consumed by the motor complex (figure 2) is manifested as mechanical power output. In the Frank *et al*. micro-chamber experiments, mechanical power output was band-pass and peaked between 30 and 70 kHz, depending on cell length. Cells continued to output significant power up to the highest frequency tested, 100 kHz. Santos-Sacchi *et al*. improved the micro-chamber approach to control membrane potential, revealing low-pass characteristics of whole-cell displacement, but mechanical power output computed from their data is similar results based on the Frank *et al*. data, and continued to increase up to the highest frequency tested, 15 kHz. Mechanical power output could not have occurred in these experiments if the imaginary component of NLC recorded membrane patches arises from dielectric loss, supporting the null hypothesis that *Im*(*C_n_*) in voltage-clamp experiments (figure 2) arises at high frequencies due to the prestin–motor complex delivering power to the viscous load.

From the electrical perspective under voltage-clamp conditions, power conversion requires negative *Im*(*C^N^*), which emerges only at high frequencies as *Re*(*C^N^*) becomes small. Although peak *Im*(*C^N^*) is smaller than peak *Re*(*C^N^*) resulting in a decline in the magnitude *Abs*(*C^N^*) with frequency, the decline does not reflect the limiting speed of motor function or a decline in electro-mechanical power conversion. Emergence of *Im*(*C^N^*) at high frequencies reveals a shift in the phase of the prestin-dependent charge displacement relative to force, which is in phase with d*v*/d*t* at low frequencies (giving rise to real NLC) and in phase with *v* at high frequencies (giving rise to negative imaginary NLC).

In summary, the present results are consistent with the hypothesis that the speed of force generation by the prestin–membrane motor complex is nearly instantaneous, while the speed of displacement is limited by the load against which the motor must deform. For maximum power output, the timing of motor charge displacement during the power stroke must be shifted −90° relative to the charge displacement measured at low frequencies in the absence of significant mechanical load. The phase shift is reflected in both electrical and mechanical aspects of the motor. Mechanical power output to a dissipative viscous load requires force to be in phase with velocity. The present report only analysed data collected under voltage-clamp commands. The extent to which high frequency power conversion draws directly from voltage-driven conformational changes in prestin versus some other mechanism is not addressed. Nevertheless, the analysis shows electro-mechanical power conversion is maximized only when voltage, current, force and velocity occur at specific phases relative to each other—requirements likely met by setting the mechano-electrical transduction current, RC corner frequency, cell stiffness, and level of prestin expression along the tonotopic map in the cochlea [37–39]. Electrical properties of the organ of Corti are also implicated as important in power conversion, through the influence of the electro-anatomy on frequency-dependent extracellular potentials and OHC transmembrane voltage [40].

## Data Availability

The manuscript includes no new original data, but includes alternative analysis of previously published raw data. New analysis methods are presented.
